# Palliative Care in Public Policy: Results from a Global Survey

**DOI:** 10.1089/pmr.2020.0062

**Published:** 2020-09-03

**Authors:** David Clelland, Danny van Steijn, Sandy Whitelaw, Stephen Connor, Carlos Centeno, David Clark

**Affiliations:** ^1^ School of Interdisciplinary Studies, University of Glasgow, Scotland, United Kingdom.; ^2^ ATLANTES Global Observatory of Palliative Care, University of Navarra, Institute for Culture and Society, Pamplona, Spain.; ^3^ Worldwide Hospice Palliative Care Alliance, London, United Kingdom.

**Keywords:** global development, mapping, palliative care, policy

## Abstract

***Background:*** Public policy has been a foundational component of the World Health Organization public health model for palliative care development since 1990. There is, however, limited evidence on the existence and character of palliative care policy at the country level.

***Objective:*** To identify, report on, and map the presence of national palliative care strategies, plans, legislation, and dedicated government resources in 198 countries.

***Design:*** An online survey generated 2017 data on indicators of national policy for palliative care.

***Subjects:*** In-country experts on palliative care.

***Measurements:*** The survey included specific questions on the existence and status of national strategies or plans, recognition of palliative care in national law, and dedicated government support.

***Results:*** Fifty-five countries have a national strategy or plan for palliative care of some sort, though levels of implementation vary. Forty-seven countries have some reference to palliative care in national law, and 24 have some form of stand-alone national law on palliative care provision or recognize it as a right in the constitution. Sixty-six countries have a dedicated section within government with responsibility for palliative care.

***Conclusions:*** There is a long way to go before palliative care around the world is universally supported by public policy intentions that will support its required development.

## Introduction

**FIG. 1.**
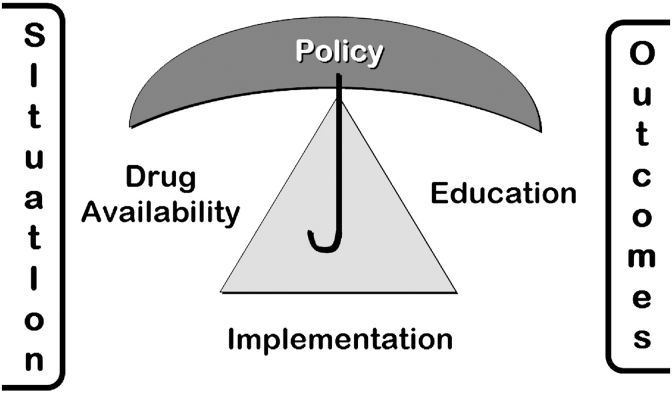
WHO public health model for palliative care development. WHO, World Health Organization.

The 2014 World Health Assembly Resolution on Palliative Care (WHA67.19) calls on all member states to develop and implement “palliative care policies to support the comprehensive strengthening of health systems to integrate evidence-based, cost-effective and equitable palliative care services in the continuum of care, across all levels, with emphasis on primary care, community and home-based care, and universal coverage schemes.”^1 (p.3)^ This emphasis on policy has been a key element of the World Health Organization (WHO) strategy to improve palliative care, from the first iteration of its three “foundation measures” (policy, education, and medicine availability) in 1990^2^ and in particular since 2007 when a fourth element (implementation) was added^3^ (Fig. 1). In this more recent model, policy is primary to the other three measures and is seen as a necessary condition for the other components to be present and effective, illustrated by the image of an umbrella where the cover represents the policy dimension, with the other dimensions below it.

In this article, we report on the existence of policies to support the development of palliative care provision at country level. In doing this, we acknowledge that policy is a multifaceted concept that can be realized in many forms, from local to global and may comprise a variety of measures and actions.^4^ We are conscious that palliative care activists often invoke the need for policies to support their goals,^5^ but we see little published evidence to demonstrate a level of policy understanding that might make their efforts more effective. Two exceptions are a forthcoming chapter in the *Oxford Textbook of Palliative Medicine*, which is helpful in mapping the policy landscape and some of its key elements as they relate to palliative care,^6^ and a recent article on the role of palliative care advocacy in inspiring and nurturing the political will necessary to support the development and funding of national palliative care policy.^7^

Our purpose in this article is pragmatic. Using data from a global survey of palliative care development,^8^ we present new evidence on the distribution and character of policies to support palliative care that were reported by in-country experts to exist in 2017. Our goal is to provide palliative care activists with new country-level data that can assist in advocacy work.

## Methods

As part of a wider assessment of global palliative care development, we conducted an online survey in 2017 of in-country palliative care experts in 198 territories, comprising the 193 Member States of the United Nations, 2 Observer States, along with Kosovo, Somaliland, and Taiwan, China. A total of 560 experts from 179 (90%) countries for which contacts could be found were asked to complete the questionnaire. We were unable to identify a contact for 19 (10%) countries. Responses to the survey were used to score each country across 10 indicators of palliative care, drawn from the emerging literature.^9^ Full results^8^ and details of the study protocol^10^ are described elsewhere.

The survey included a number of questions about the existence of strategies, legislation, and resources that could be perceived as evidence of policies in support of palliative care. Our analysis here, therefore, focuses on these questions, where participants were asked whether their country had any of the following:

- A national strategy or plan specific to palliative care, one that has been implemented and is regularly evaluated, and one that has been updated.
- A reference to palliative care in national law.
- A specific (stand-alone) palliative care law or recognition of palliative care as a right in the constitution.
- A person/desk/unit in a government department with palliative care responsibility.

Participants were given the option to respond to these items with yes, no, in progress, or don't know. Where countries had multiple conflicting responses to a question that could not be resolved, these were classified as such in the reporting of the results.

## Results

### National palliative care strategies

**FIG. 2.**
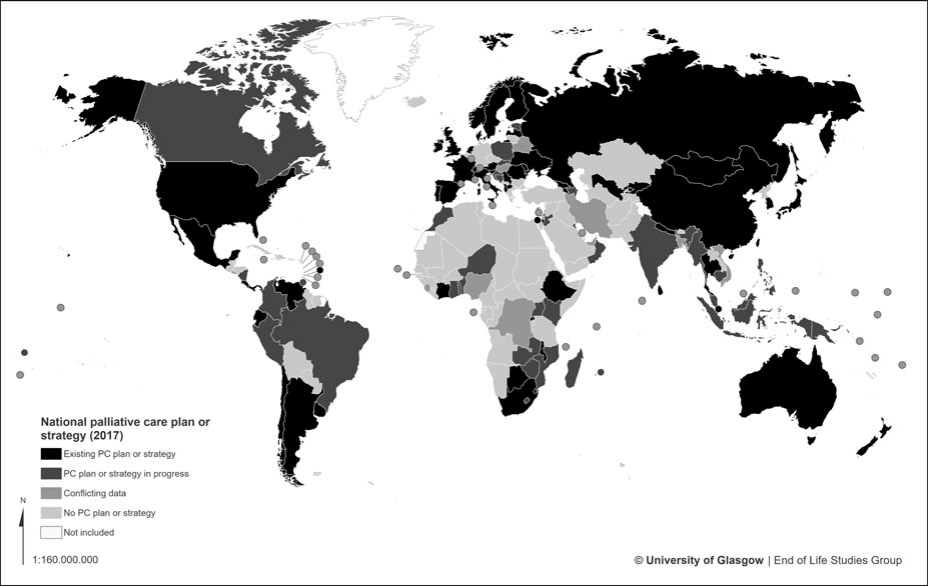
World map of national palliative care strategy development.

Some form of dedicated national palliative care strategy or plan exists in 55 countries, just over a quarter (28%) of the global total. A further 36 countries do not have a national strategy in place but report making progress toward this (Fig. 2 and Table 1).

**Table 1.** National Palliative Care Strategies or Plans

Status	Countries
A national strategy or plan specific to palliative care (55 countries)	Albania, Argentina, Armenia, Australia, Austria, Barbados, Botswana, Denmark, Chile, China, Côte d'Ivoire, Costa Rica, Ecuador, Ethiopia, Finland, France, Georgia, Ireland, Israel, Italy, Japan, Kyrgyzstan, Latvia, Macedonia, Malawi, Mexico, Moldova, Mongolia, Nepal, Netherlands, New Zealand, Norway, Panama, Portugal, Romania, Russia, Rwanda, Serbia, Singapore, Slovenia, South Africa, South Korea, Spain, Sri Lanka, Swaziland, Switzerland, Taiwan, Thailand, Sweden, Ukraine, United Kingdom, Uruguay, United States, Uzbekistan, Venezuela,
In progress (36)	Azerbaijan, Benin, Bosnia and Herzegovina, Brazil, Cambodia, Canada, Colombia, Croatia, Estonia, Ghana, India, Indonesia, Kenya, Kuwait, Lebanon, Lesotho, Madagascar, Malaysia, Mauritius, Morocco, Mozambique, Myanmar, Nicaragua, Niger, Oman, Papua New Guinea, Peru, Philippines, Poland, Samoa, Slovakia, Togo, Trinidad and Tobago, Uganda, Zambia, Zimbabwe
No national strategy or plan (36)	Bahamas, Bolivia, Bulgaria, Burundi, Cameroon, Czech Republic, Dominican Republic, Egypt, Eritrea, Fiji, Germany, Greece, Guatemala, Guinea, Haiti, Honduras, Iceland, Iraq, Jamaica, Kazakhstan, Liberia, Libya, Liechtenstein, Lithuania, Luxembourg, Malta, Mauritania, Pakistan, Palestine, Paraguay, Saudi Arabia, Senegal, South Sudan, Sudan, Tajikistan, Tunisia
No data (59)	Afghanistan, Algeria, Andorra, Angola, Antigua and Barbuda, Bahrain, Belgium, Belize, Bhutan, Brunei, Burkina Faso, Cape Verde, Central African Republic, Chad, Comoros, Congo(Republic), Cuba, Djibouti, Dominica, Equatorial Guinea, Gabon, Gambia, Grenada, Guinea-Bissau, Guyana, Kiribati, Kosovo, Laos, Maldives, Mali, Marshall Islands, Micronesia, Monaco, Montenegro, Namibia, Nauru, North Korea, Palau, Qatar, Saint Lucia, San Marino, Sao Tome e Principe, Seychelles, Solomon Islands, Somalia, Somaliland, St. Kitts and Nevis, St. Vincent and the Grenadines, Suriname, Syria, Tanzania, Timor l'Este, Tonga, Turkey, Turkmenistan, Tuvalu, Vanuatu, Vatican City, Yemen
Conflicting data (12)	Bangladesh, Belarus, Congo (DR), Cyprus, El Salvador, Hungary, Iran, Jordan, Nigeria, Sierra Leone, United Arab Emirates, Vietnam

### Palliative care in national law

Forty-seven countries (24% of those surveyed) have a reference to palliative care within a specific national law, with another 12 (6%) reporting some progress toward this.

**FIG. 3.**
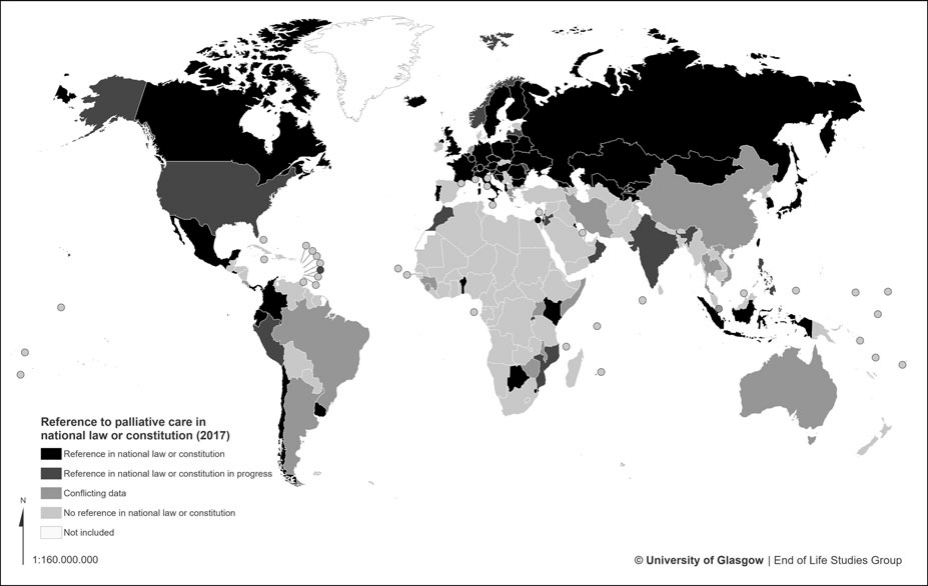
World map of palliative care in national law.

Twenty-four countries (12%) have a specific stand-alone palliative care law, or recognition of palliative care as a right in the national constitution. Again, however, an additional eight countries (4%) report making progress in this direction (Fig. 3 and Table 2).

**Table 2.** Countries with Palliative Care in National Law

Status	Countries
A reference to palliative care in a national law (47)	Albania, Austria, Belarus, Belgium, Benin, Bosnia and Herzegovina, Bulgaria, Canada, Chile, Colombia, Croatia, Czech Republic, Ecuador, El Salvador, Finland, France, Georgia, Germany, Hungary, Iceland, Indonesia, Israel, Italy, Japan, Kazakhstan, Kenya, Kyrgyzstan, Liechtenstein, Lithuania, Luxembourg, Mexico, Mongolia, Panama, Poland, Portugal, Romania, Russia, Slovenia, South Korea, Sweden, Switzerland, Taiwan, Tajikistan, Ukraine, United Kingdom, Uruguay, Uzbekistan
In progress (12)	Barbados, Botswana, India, Jordan, Latvia, Moldova, Morocco, Norway, Peru, Philippines, Rwanda, United States
Conflicting data (15)	Argentina, Armenia, Australia, China, Costa Rica, Greece, Iran, Lebanon, Malawi, Netherlands, Singapore, Thailand, Uganda, Vietnam, Zimbabwe
A specific (stand-alone) palliative care law or recognition of palliative care as a right in the constitution (24)	Albania, Belgium, Botswana, Canada, Colombia, Croatia, France, Germany, Indonesia, Israel, Italy, Japan, Kenya, Kuwait, Luxembourg, Mexico, Mongolia, Poland, Portugal, Romania, Slovenia, South Korea, Swaziland, Taiwan
In progress (8)	Bosnia and Herzegovina, Finland, India, Latvia, Mozambique, Oman, Russia, Uzbekistan
Conflicting (13)	Brazil, China, Costa Rica, Guinea, Hungary, Jordan, Lebanon, Malawi, Rwanda, Sierra Leone, Uganda, Vietnam, Zimbabwe

### Dedicated government resource to support palliative care

**FIG. 4.**
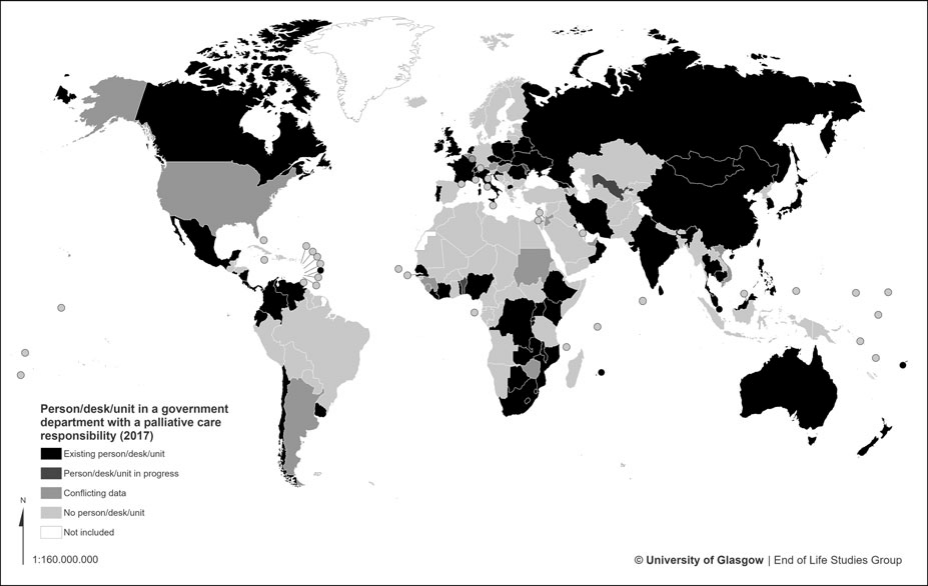
World map of government palliative care resource.

The existence of a dedicated person or unit within the national government department with responsibility for palliative care is reported in 66 countries, a third (33%) of the global total. In three countries work toward this is underway. Responses from a further 15 countries provide conflicting assessments as to whether or not this support exists (Fig. 4 and Table 3).

**Table 3.** Countries with a Person/Desk/Unit in a Government Department with Palliative Care Responsibility

Status	Country
Yes (66)	Australia, Barbados, Belarus, Botswana, Burundi, Cambodia, Canada, Chile, China, Colombia. Costa Rica, Côte d'Ivoire, Croatia, Czech Republic, Ecuador, El Salvador, Ethiopia, Fiji, France, Georgia, India, Iran, Ireland, Italy, Japan, Kenya, Kuwait, Lesotho, Liberia, Macedonia, Malawi, Malaysia, Mauritius, Mongolia, Mozambique, Netherlands, New Zealand, Nicaragua, Nigeria, Oman, Panama, Philippines, Poland, Portugal, Romania, Russia, Rwanda, Senegal, Singapore, Slovakia, South Africa, South Korea, Sri Lanka, Swaziland, Switzerland, Taiwan, Thailand, Togo, Uganda, Ukraine, United Kingdom, Uruguay, Venezuela, Zambia, Congo (DR), Mexico,
In progress (3)	Benin, Moldova, Uzbekistan
Conflicting (15)	Argentina, Armenia, Austria, Belgium, Cyprus, Guinea, Hungary, Jordan, Luxembourg, Sierra Leone, Sudan, United Arab Emirates, United States, Vietnam, Zimbabwe
No person/desk/unit (56)	Albania, Azerbaijan, Bahamas, Bahrain, Bangladesh, Bolivia, Bosnia and Herzegovina, Brazil, Bulgaria, Cameroon, Denmark, Dominican Republic, Egypt, Eritrea, Estonia, Finland, Germany, Ghana, Greece, Grenada, Guatemala, Haiti, Honduras, Iceland, Indonesia, Iraq, Israel, Jamaica, Kazakhstan, Kyrgyzstan, Latvia, Lebanon, Libya, Liechtenstein, Lithuania, Madagascar, Malta, Mauritania, Morocco, Myanmar, Nepal, Norway, Pakistan, Palestine, Papua New Guinea, Paraguay, Peru, Samoa, Saudi Arabia, Serbia, South Sudan, Spain, Sweden, Tajikistan, Trinidad and Tobago, Tunisia
No data (58)	Afghanistan, Algeria, Andorra, Angola, Antigua and Barbuda, Belize, Bhutan, Brunei, Burkina Faso, Cape Verde, Central African Republic, Chad, Comoros, Congo (Republic), Cuba, Djibouti, Dominica, Equatorial Guinea, Gabon, Gambia, Guinea-Bissau, Guyana, Kiribati, Kosovo, Laos, Maldives, Mali, Marshall Islands, Micronesia, Monaco, Montenegro, Namibia, Nauru, Niger, North Korea, Palau, Qatar, Saint Lucia, San Marino, Sao Tome e Principe, Seychelles, Slovenia, Solomon Islands, Somalia, Somaliland, St. Kitts and Nevis, St. Vincent and the Grenadines, Suriname, Syria, Tanzania, Timor l'Este, Tonga, Turkey, Turkmenistan, Tuvalu, Vanuatu, Vatican City, Yemen

### Characteristics of countries with developed support for palliative care policy

Comparing these results with the World Bank classification of national income level^11^ demonstrates that the majority of countries with an established national palliative care strategy, reference to palliative care in a national law, or a dedicated individual or unit within government responsible for palliative care are in the high- or upper–middle-income categories. Nevertheless, there are also a number of relatively poor countries with these elements in place (Table 4).

**Table 4.** Countries with Palliative Care Policy by World Bank Income Level

	Low	Lower middle	Upper middle	High	Total
National strategy or plan specific to palliative care	4	9	14	28	55
Reference to palliative care in national law	2	8	10	27	47
Person/desk/unit in a government department	10	15	17	24	66

### Limitations

The wider study from which these results were drawn represents the most comprehensive attempt yet to map the global development of palliative care, and the latest step in an iterative process that has gradually produced more robust and comprehensive methods of measurement. Nevertheless, the method still has certain limitations and we have described these elsewhere.^10^

More specifically, for the items described here, we encountered some practical limitations in assessing measures relating to policy. First, our findings on the various policy-related indicators have not been verified with documentary sources. Second, where countries have more decentralized systems of governance or high levels of regional autonomy related to health care provision, a focus on national policies may conceal significant within-country variation in the development of palliative care policy. Third, some specific items we used may provide only limited insights. For example, the item concerning the presence of a government resource devoted to palliative care sets a relatively low bar, as a question with a binary choice to which respondents could answer “yes” even if they had identified only a single government employee, perhaps with palliative care as only one responsibility among many. Furthermore, although a dedicated government desk might represent a significant level of resource in a small country, this would be less true in larger countries; a more sensitive measure would generate more valuable data in this instance.

## Discussion

As our results show, and despite WHO endorsement, national policy recognition for palliative care is far from universal and is generally (though not exclusively) confined to high-income countries. There is some evidence, however, that a significant number of others, including low- and middle-income countries, are making progress in this direction. The long-term impact of the Open Society Foundations initiative on palliative care in Central and Eastern Europe can perhaps be seen in our data.^12^

A 2015 WHO survey reported that 37% of countries had an operational national policy for noncommunicable diseases that included palliative care.^13^ This is a higher figure than we report here for the number of countries with a stand-alone policy. Two factors may explain the difference. First the WHO study looked for the inclusion of palliative care within a wider policy commitment, whereas we focused on stand-alone plans or strategies for palliative care. Second, the WHO study drew on reports from government officials (who perhaps might be inclined to inflate levels of attention to palliative care), whereas our main sources were palliative care activists and experts (perhaps inclined to underestimate or downplay the presence of such policies). We recognize that methodological issues of this kind are still to be fully overcome if we are to generate more accurate information on many aspects of global palliative care development. There are also wider more conceptual challenges.

The creation and implementation of palliative care policy face specific psychological, political, financial, and social barriers.^6^ For example, in their review of palliative care policy in Ireland, a country recognized as something of a role model in this regard,^14^ May et al.^15^ nevertheless identify the level of resource required, and competition for this resource from other policy areas, as having inhibited the delivery of ambitions for palliative care expansion. Likewise, noting the long-standing interest in a “policy-implementation gap”^16^ across a variety of public policy areas, Hudson et al.^17^ identify four factors that prevent policy from achieving its stated aims: overly optimistic expectations, problems of implementation in systems of dispersed governance, inadequate collaborative policymaking, and the political cycle itself. From this point of view, the existence, for example, of a national law or strategy, may be an indicator of recognition, but not itself a practical step to securing provision. This recognition of policies as a *necessary* but not *sufficient* condition for implementation is indeed acknowledged by the 2015 WHO study:

Explicit national policies for palliative care provide a necessary foundation for palliative care development, but successful policies require universal essential palliative medicine accessibility, routine education of health care workers in palliative care, and widespread implementation.^13^

Such actions are also likely to require a costed national plan, with political and financial commitment over several decades.^6^ A key question is how to influence the development and implementation of policy, and where, for global activists, the balance of their limited resources should rest.

How then should “policy” be understood in the measurement of palliative care development? The updated WHO model, although acknowledging that they must be addressed in a coordinated way,^18^ accommodates policy alongside other foundation measures (education, medicine availability, and implementation). Accordingly, we included indicators in this study relating to strategy, relevant laws, and government coordination and support. Future studies need to refine these indicators, but also recognize that there is not necessarily a linear process leading from policy goals to specific interventions and then to outcome,^19^ not least as palliative care development in many countries continues to be spearheaded by motivated individuals and nongovernment organizations, often with limited policy influence.^20^ There may be scope for future research in this area to explore perceptions of the effectiveness of policies in advancing provision in different national contexts.

Conceptual work is continuing to refine the detailed dimensions that can make up an indicator of palliative care policy. A *Brief Manual on Health Indicators Monitoring Global Palliative Care Development*,^21^ published after the present study was designed, and supported by a systematic review,^9^ contains eight subindicators within the policy domain, each with a clear definition. These would merit a separate research study at the global level.

## Conclusions

Within the growing literature on the global development of palliative care, there is a significant strand that sees policy as an important element. Even allowing for the limitations of our study, it is clear that there is a long way to go before palliative care is universally supported by policy intentions that can lead to demonstrable outcomes:

- Only 55 countries globally have a national strategy or plan for palliative care and not all of these have implementation with evaluation and updating.
- A further 37 countries are making progress toward the establishment of a national strategy.
- Forty-seven countries have some reference to palliative care in national law, and 24 have some form of stand-alone national law on palliative care provision or recognize it as a right in the constitution.
- Sixty-six countries have a dedicated section within government with responsibility for palliative care.

These figures will be disappointing to many who have sought to use the WHO model as their guide over the past 30 years. Our findings point to the need for greater clarity on which sets of actions and indicators comprise a “policy response” to the need for palliative care. Alongside this it would be helpful to consider in depth the experience in countries that have been successful in building up policy capacity relating to palliative care, the factors that have shaped their success, and how the lessons learned can effectively be transferred and translated elsewhere.

## Abbreviation Used

WHO: World Health Organization
